# Prevalence of and Risk Factors Associated with Polycystic Ovary Syndrome Among Female University Students of Health Sciences in a Middle Eastern Country

**DOI:** 10.1089/whr.2023.0176

**Published:** 2024-07-31

**Authors:** Fay Alkhezi, Nourah AlNemash, Joud AlMutairi, Sarah Saleh, Mariam AlMutairi, Shahad Saleh, Saeed Akhtar

**Affiliations:** Department of Community Medicine and Behavioural Sciences, College of Medicine, Kuwait University, Kuwait.

**Keywords:** polycystic ovary syndrome, prevalence, risk factors, young adult university students

## Abstract

**Background:**

Polycystic ovary syndrome (PCOS) is one of the most common gynecological endocrinopathies in women of reproductive age in the Middle Eastern countries, including Kuwait. This cross-sectional study assessed the prevalence of and examined the factors associated with PCOS status among female university students of health sciences in Kuwait.

**Methods:**

During January 2023, a cross-sectional study was conducted among female students, enrolled in any of the five colleges (i.e., Medicine, Public Health, Dentistry, Pharmacy, and Allied Health Sciences) of Kuwait University. Data were collected using a structured e-questionnaire administered through social media platforms. The prevalence (%) of physician-diagnosed PCOS was computed. The multivariable logistic regression model was used to compute the adjusted odds ratios (aOR) and corresponding 95% confidence intervals (CI) for the factors significantly associated with PCOS status.

**Results:**

Of the 588 participants, most were Kuwaiti (86.5%), single (95.6%), and 21–24 years old (59.3%). The prevalence of PCOS was 16.3% (96/588). The risk factors significantly associated with PCOS status were hyperprolactinemia (aOR = 7.67; 95% CI: 3.72–15.83), menstrual irregularities (aOR = 5.12; 95% CI: 2.32–11.31), family history of PCOS (aOR = 3.49; 95% CI: 1.93– 6.29), hirsutism (aOR = 3.58; 95% CI: 2.06–6.21), and male pattern baldness (aOR = 2.06; 95% CI: 1.19–3.58).

**Conclusions:**

This study showed moderately high prevalence of PCOS. Hyperprolactinemia, menstrual irregularities, family history of PCOS, hirsutism and baldness were significantly associated with PCOS status in the study sample. Imparting awareness and early diagnosis may help minimize PCOS burden in this and other similar settings in the region. If implemented, future studies may look at the impact of such efforts.

## Introduction

Polycystic ovary syndrome (PCOS) is the most common endocrine disorder among women of reproductive age, defined as a hormonal imbalance with at least one polycystic ovary accompanied by ovulatory dysfunction and excessive secretion of androgens.^1,2^ The etiology of PCOS is multifactorial in which both genes and the environment (e.g., obesity and physical inactivity) play a role in its causation. The reproductive and metabolic features of PCOS are somewhat reversible with lifestyle modifications.^3^ The high prevalence of PCOS along with its serious long-term health consequences makes PCOS a public health concern. Reportedly, by the age of 30 years, ∼25%–30% of women with PCOS show impaired glucose tolerance and 8% of affected women tend to develop type 2 diabetes mellitus annually with an increased risk of hypertension. In addition, the chronic anovulation predisposes the women to increased infertility and endometrial cancer risk.^4^

The World Health Organization (WHO) data suggest that globally, ∼116 million (3.4%) women are affected by PCOS.^5^ A review of ethnic comparison of relative susceptibility to PCOS across four ethic groups, i.e., Middle Eastern, Caucasian, African American, and Chinese, revealed the highest PCOS prevalence among the Middle Eastern women (16%, Rotterdam criterion) followed by the Chinese women (5.6%, Rotterdam criterion) compared with the other groups.^6^ In addition, a meta-analysis showed that PCOS prevalence in the Middle Eastern populations is 11.9%, with relatively higher PCOS prevalence of 18.8% among women in the Gulf region.^7^ Another study among female university students in Palestine reported 7.3% PCOS prevalence.^8^

The review of risk factors showed that physical inactivity, abnormal menstrual cycle, and overweight/obesity (i.e., waist–hip ratio > 0.85) are known risk factors for PCOS.^9,10^ However, it has been shown that the lifestyle changes and control of weight tend to reduce the symptoms of PCOS and help reverse the hormonal imbalance that leads to the development of PCOS.^11^ There is a lack of sufficient published data on the magnitude of the burden and risk factors for PCOS in various countries in the Middle East, particularly in Kuwait. Therefore, this cross-sectional study sought to assess the prevalence of and the factors associated with PCOS status among female students enrolled in five health sciences colleges of Kuwait University.

## Methods

### Study design, setting, and participants

During January 2023, a cross-sectional study was conducted to assess the prevalence of and examine the risk factors for PCOS among young adult females (≥18 years). The female students of any nationality (Kuwaiti and non-Kuwaiti), enrolled in any of the five health sciences colleges (i.e., Medicine, Public Health, Dentistry, Pharmacy, and Allied Health Sciences) at the Health Sciences Center, Kuwait University, were eligible for inclusion. A structured and self-administered questionnaire was used to collect the data from the enrolled participants.

### Questionnaire

A structured questionnaire was developed in both English and Arabic. It included a total of 29 questions composed of dichotomous (yes/no), as well as multiple-choice questions. The questionnaire took on average around 10 minutes for its completion. The questionnaire comprised three sections as summarized below.

#### Sociodemographic variables

This section comprised of questions on age-group (18–20 years, 21–24 years, and 25 years old or older), nationality (Kuwaiti or non-Kuwaiti), marital status (never married, ever married), and their major academic field (medicine, dentistry, pharmacy, allied health science, and public health).

#### PCOS assessment

In this section, the participants were asked whether they were diagnosed with PCOS by a physician. If they were diagnosed with PCOS, their age at diagnosis and whether they received treatment were enquired. To rule out other related conditions, and for a differential diagnosis of PCOS, questions were asked about the diagnosis of hypothyroidism and hyperprolactinemia. We assessed the symptoms typical of PCOS according to the criteria of the National Institutes of Health (NIH), USA, including menstrual irregularity and hyperandrogenism, hirsutism (the location and severity of thick hair growth), acne (five or more pimples), and male pattern baldness.^1,12^

#### Risk factors for PCOS

Questions on the potential risk factors for PCOS such as height (cm), weight (kg), had trouble in losing weight and any discoloration in body folds (underarms, groin, or neck) were included. In addition, questions about the family history of PCOS, menstrual irregularities, and diabetes were included. If the family member(s) reportedly had one or more of these ailments, the relationship of the respondent with the family member was enquired. In addition, the participants were asked whether they were diagnosed with type 1 or type 2 diabetes by a physician.

### Sampling, sample size, and data collection

As noted earlier, the population of interest was female students enrolled in any of the five colleges of the Health Sciences Center, Kuwait University, of any nationality (Kuwaiti and non-Kuwaiti). Data were collected from January 2–5, 2023, from all the five colleges of the Health Sciences Center, Kuwait University, by three data collection teams of six 5th year medical students (two members per team). One team collected the data from the College of Medicine, and the other two teams collected the data from the remaining four colleges (two colleges per team). Overall, we intended to enroll about 600 students. This sample size was proportionally allocated to the total enrollment in each of the five colleges. The classes’ schedules were obtained from the office of the vice dean for students’ affairs of each college. The research members took the permission from the lecturers of each class, and the questionnaire was distributed via QR code at the beginning or the end of the class, and they waited for the students to fill-in the questionnaire, which took them on average 10 minutes.

### Ethics

The research protocol and data collection instrument were approved by the Kuwait University, Health Sciences Center Ethics Committee as well as the Ethics Committee of the Ministry of Health in Kuwait. The questionnaire included a consent form that explained the purpose of the study and clarified that participation is completely voluntary. It also stated that personal information was not required, and data collection was anonymous to ensure the confidentiality of collected data. Moreover, no negative consequences would result if the prospective participant does not choose to participate.

### Data management and statistical analysis

Data were gathered using Google Forms and converted to a Microsoft Excel file and then into the SPSS file for analysis. We reviewed and cleaned the datafile and excluded errors and inadmissible answers before starting the data analysis. To characterize the study sample, descriptive statistics of sociodemographics, including mean (SD) and proportion (%) as appropriate, were computed. Prevalences of PCOS, both physician-diagnosed and self-reported PCOS, were computed. For each participant, the outcome variable PCOS status was defined as an affirmative PCOS (coded = 1; if diagnosed by a physician and/or self-reported) and PCOS negative otherwise (coded = 0). Chi-squared analysis was used to test the statistical significance of the association of demographics and hypothesized risk factors with PCOS status. The variables significantly (*p* ≤ 0.150) related to PCOS status were further subjected to univariable and multivariable logistic regression analyses. For the selection of final multivariable logistic regression model, backward stepwise procedure was used, and the variables significantly (*p* < 0.05) associated with PCOS status were retained in the final model. Adjusted odds ratios (aOR) and their corresponding 95% confidence intervals (CI) were derived from the model coefficients and their standard errors for the variables in the final model and used for the interpretation of the results.

## Results

### Sociodemographic characteristics

The total enrollment for the 2nd semester of 2022–23 academic year in each college was 783, 202, 360, 1142, and 122 students in the College of Medicine, Dentistry, Pharmacy, Allied Health Sciences, and Public Health, respectively. We enrolled 222, 44, 92, 201, and 29 students, respectively, from each of the colleges. Thus, a total of 588 female students from five Colleges of Health Science Center, Kuwait University, consented and completed the questionnaire. In this sample, 36.4% of participants were aged between 18 and 20 years, 59.3% were between 21 and 24 years of age, and 4.2% were 25 years old or older. Most participants were Kuwaiti (86.5%) and single (95.6%). The study participants were from College of Medicine (222, 36.8%), College of Allied Health Sciences (201, 34.2%), College of Pharmacy (92, 15.6%), College of Dentistry (44, 7.5%), and College of Public Health (29, 4.8%) (Table 1).

**Table 1.** Sociodemographic Characteristics of Female University Students of Five Colleges of Health Sciences Center, Kuwait University, Enrolled in a Cross-Sectional Study of Prevalence and Risk Factors for Polycystic Ovary Syndrome, December 2022 (*n* = 588)

Variable	*n*	%
Age (completed years)		
18–20	214	36.4
21–24	349	59.3
≥25	25	4.2
Nationality		
Kuwaiti	526	86.5
Non-Kuwaiti	62	10.2
Marital status		
Single	562	95.6
Married	26	4.4
College of study		
Medicine	222	36.8
Dentistry	44	7.5
Pharmacy	92	15.6
Allied Health Sciences	201	34.2
Public health	29	4.8

### Distribution of PCOS-related variables

The prevalence of PCOS among female students at Colleges of Health Sciences Center who were diagnosed by a physician was 16.3% (96/588), and 11.7% of them received treatment. Of the participants, 7.8% were diagnosed with hypothyroidism and 8.5% with hyperprolactinemia. Reportedly, 36.7% of the participants experienced infrequent menstruation, and 25.2% experienced frequent menstruation. The distributions of other PCOS-related variables including hirsutism (32.3%), acne experienced (74.0%), baldness experienced (26.4%), difficulty in losing weight (43.0%), and discoloration (39.6%) are presented in Table 2.

**Table 2.** Distribution of Polycystic Ovary Syndrome–Related Variables in the Study Sample of Female Students of Various Colleges of Health Sciences Center, Kuwait University Enrolled in a Cross-Sectional Study, Kuwait, December 2022 (*n* = 588)

PCOS-related health variables	*n*	%
PCOS diagnosis by physician		
Yes	96	16.3
No	492	83.7
PCOS treatment		
Yes	69	11.7
No	294	50.0
Hypothyroidism		
Yes	46	7.8
No	542	92.2
Hyperprolactinemia		
Yes	50	8.5
No	538	91.5
Menses irregularities		
Infrequent menstruation	216	36.7
Frequent menstruation	148	25.2
Normal	224	38.1
Hirsutism		
Yes	190	32.3
No	398	67.7
Acne experienced		
Yes	435	74.0
No	153	26.0
Baldness experienced		
Yes	155	26.4
No	433	73.6
Difficulty in losing weight		
Yes	253	43.0
No	335	57.0
Discoloration		
Yes	233	39.6
No	355	60.4

PCOS, polycystic ovary syndrome.

### Chi-squared analysis of potential risk factors for their association with PCOS status

The sociodemographic characteristics of the participants and PCOS-related variables which were significantly associated with PCOS status on chi-squared analysis included the age (*p* = 0.028), experiencing hyperprolactinemia (*p* < 0.001), menstrual irregularities (*p* < 0.001), hirsutism (*p* < 0.001), acne (*p* = 0.043), baldness (*p* < 0.001), difficulty in losing weight (*p* < 0.001), discoloration (*p* < 0.001) and body mass index (*p* = 0.002). Moreover, other significant risk factors on chi-squared analysis were the family history of PCOS (*p* < 0.001) and the family history of menstrual irregularities (*p* < 0.001) (Table 3).

**Table 3.** Chi-Squared Analysis of Potential Risk Factors for Polycystic Ovary Syndrome in a Study Sample of Female Students from Five Colleges of Health Sciences Center, Kuwait University, Enrolled in a Cross-Sectional Study, Kuwait, December (*n* = 588)

Predictors	PCOS = 96 (16.3%) *n* (%)	No PCOS = 492 (83.7%) *n* (%)	*p*-Value
Age (years)			0.612
18–24	91 (16.2)	472 (83.8)	
≥25	5 (20.0)	20 (80.0)	
Nationality			0.856
Kuwaiti	87 (16.5)	439 (83.5)	
Non-Kuwait	9 (14.5)	53 (85.5)	
Marital status			0.411
Single	90 (16.0)	472 (84.0)	
Married	6 (23.1)	20 (76.9)	
College of study			0.656
Medicine	34 (15.3)	188 (84.7)	
Dentistry	9 (20.5)	35 (79.5)	
Pharmacy	13 (14.1)	79 (85.9)	
Allied Health Science	37 (18.4)	164 (81.6)	
Public Health	3 (10.3)	26 (89.9)	
Hypothyroidism			0.149
Yes	11 (23.9)	35 (76.1)	
No	85 (15.7)	457 (84.3)	
Hyperprolactinaemia			<0.001
Yes	33 (66.0)	17 (34.0)	
No	63 (11.7)	457 (88.3)	
Menstruation irregularities			<0.001
Yes	88 (24.2)	276 (75.8)	
No	8 (3.6)	216 (96.4)	
Hirsutism			<0.001
Yes	67 (35.3)	123 (64.7)	
No	29 (7.3)	369 (92.7)	
Acne			0.043
Yes	79 (18.2)	356 (81.8)	
No	29 (7.3)	136 (88.9)	
Baldness			<0.001
Yes	48 (31.0)	107 (69.0)	
No	48 (11.1)	385 (88.9)	
Difficulty in losing weight			<0.001
Yes	58 (22.9)	195 (77.1)	
No	38 (11.3)	297 (88.7)	
Discoloration			<0.001
Yes	57 (24.5)	176 (75.5)	
No	39 (11.0)	316 (89.0)	
PCOS family history			<0.001
Yes	74 (26.3)	207 (73.7)	
No	22 (7.4)	285 (92.8)	
Family history menstruation irregularities			0.002
Yes	72 (20.1)	286 (79.9)	
No	24 (10.4)	206 (89.6)	
Family history of diabetes			0.380
Yes	66 (15.4)	362 (84.6)	
No	30 (18.8)	130 (81.2)	
Physical exercise			0.422
Neve	49 (15.8)	262 (84.2)	
1–2 times/week	37 (18.2)	66 (81.8)	
3–5 times/week	7 (13.2)	46 (86.8)	
Almost daily	3 (14.3)	18 (85.7)	
Type 1 diabetes			0.584
Yes	3 (21.4)	11 (78.6)	
No	93 (16.4)	481 (83.8)	
Type 2 diabetes			0.058
Yes	3 (50)	3 (50)	
No	93 (16)	489 (84)	
BMI (mean ± SD)	25.78 ± 5.7	23.79 ± 4.7	0.002

BMI, body mass index; SD, standard deviation.

### Univariable logistic regression analysis of potential risk factors of PCOS

Univariable logistic regression analysis showed that the students were significantly more likely to have PCOS, if they experienced hyperprolactinemia (unadjusted OR =14.6; CI: 7.71–27.80; *p* < 0.001), menstrual irregularity (unadjusted OR = 8.61; CI: 4.09–18.14; *p* < 0.001), hirsutism (unadjusted OR = 6.93; CI: 4.26 −11.21; *p* < 0.001), acne experience (unadjusted OR =1.78; CI: 1.01–3.11; *p* = 0.0.045), acne grading 5 or more pimples (unadjusted OR =1.75; CI: 1.06–2.87; *p* = 0.028), acne diagnosis (unadjusted OR = 1.97; CI: 1.26–3.07; *p* = 0.003), baldness (unadjusted OR = 3.60; CI: 2.29–5.67; *p* < 0.001), difficulty in losing weight (unadjusted OR = 2.33; CI: 1.49–3.64; *p* < 0.001), discoloration of various body parts (unadjusted OR = 2.62; CI: 1.68–4.10; *p* < 0.001), family history of PCOS (unadjusted OR = 4.63; CI: 2.79–7.70; *p* < 0.001) and type 2 diabetes (unadjusted OR = 5.26; CI: 1.05–26.45; *p* = 0.044) (Table 4).

**Table 4.** Univariable Logistic Regression Analysis of Various Risk Factors for Polycystic Ovary Syndrome in a Sample of Female Students from Five Colleges of Health Sciences Center, Kuwait University, Enrolled in a Cross-Sectional Study, Kuwait, December 2022, (*n* = 588)

Predictors of PCOS	Unadjusted OR (95% CI)	*p*-Value
Age (completed years) (18–24 vs. ≥25)	0.77 (0.28–2.11)	0.612
Hypothyroidism (yes vs. no)	1.69 (0.82–3.45)	0.151
Hyperprolactinemia (yes vs. no)	14.64 (7.71–27.80)	<0.001
Menstrual irregularity (yes vs. no)	8.61 (4.09–18.14)	<0.001
Hirsutism (yes vs. no)	6.93 (4.28–11.21)	<0.001
Acne experienced (yes vs. no)	1.78 (1.01–3.11)	0.045
Acne grading (≥ 5 pimples) (yes vs. no)	1.75 (1.06 – 2.87)	0.028
Acne diagnosis (yes vs. no)	1.97 (1.26–3.07)	0.003
Baldness (yes vs. no)	3.60 (2.29-5.67)	<0.001
Difficulty in losing weight (yes vs. no)	2.33 (1.49–3.64)	<0.001
Discoloration of various body parts	2.62 (1.68–4.10)	<0.001
PCOS family history (yes vs. no)	4.63 (2.79–7.70)	<0.001
Type 2 diabetes mellitus (yes vs. no)	5.26 (1.05–26.45)	0.044

### Multivariable logistic regression model

Multivariable logistic regression model of the risk factors for PCOS showed that the respondents have had significantly higher odds of having PCOS diagnosis, if reportedly they have had hyperprolactinemia (aOR = 7.67; 95% CI: 3.72–15.83, *p* < 0.001), menstrual irregularities (aOR = 5.12; CI: 2.32–11.31, *p* < 0.001), family history of PCOS (aOR = 3.49; 95% CI: 1.93–6.29, *p* < 0.001), hirsutism (aOR = 3.58; 95% CI: 2.06–6.21, *p* < 0.001), or male-pattern baldness (aOR = 2.06; 95% CI: 1.19–3.58, *p* = 0.010) (Table 5).

**Table 5.** Multivariable Logistic Regression Model of Risk Factors for Polycystic Ovary Syndrome in a Sample of Female Students from Five Colleges of Health Sciences Center, Kuwait University, Enrolled in a Cross-Sectional Study, Kuwait, December 2022 (*n* = 588)

Predictors of polycystic ovary syndrome (PCOS)	Adjusted odds ratio (95% confidence interval)	*p*-Value
Hyperprolactinemia (yes vs. no)	7.67 (3.72–15.83)	<0.001
Menstrual irregularity (yes vs. no)	5.12 (2.32–11.31)	<0.001
Family history of PCOS (yes vs. no)	3.49 (1.93–6.29)	<0.001
Hirsutism (yes vs. no)	3.58 (2.06–6.21)	<0.001
Baldness (yes vs. no)	2.06 (1.19–3.58)	0.010

## Discussion

The aim of this study was to assess the prevalence of PCOS among female university students of health sciences in Kuwait and examine the risk factors for this disease in the study sample. For this purpose, female students of health sciences from Kuwait University were enrolled, and information was sought through a self-administered questionnaire.

The prevalence of physician-diagnosed PCOS among female students at the Colleges of Health Sciences Center was 16.3% in the study. This result coincides with the findings of a study conducted by Ding and colleagues, which found that the prevalence of PCOS was 16% among the Middle Eastern women.^6^ Relatively recently, a study from Saudi Arabia reported a 16% PCOS prevalence among female (21–25 years old) university students.^13^ This consistency in results may be due to the usage of similar methods in both the studies, that is, use of a self-administered questionnaire to assess the prevalence of PCOS in female university students. Furthermore, it seems to be the result of similar geographical locations and lifestyle factors in both Kuwait and Saudi Arabia. Another study prospectively estimated the prevalence of PCOS by selecting women in an unbiased manner by using data from 154 consecutive Caucasian blood donors at a hospital in Madrid, Spain. Using the NIH diagnostic criteria, the study found the PCOS prevalence as 6.5%.^14^ In addition, a previous study conducted a review of ethnic comparisons of relative susceptibility to PCOS across four ethnic groups, i.e., Middle Eastern, Caucasian, African American, Chinese, and showed that the prevalence of PCOS was the highest among African American (7.4%), followed by the Middle Eastern women (5.6%) assessed by using the NIH criteria.^15^ Two studies from Iran reported PCOS prevalence as 11.7%^16^ and 7%,^17^ assessed based on the NIH criteria. The PCOS prevalence (based on the NIH standards) in a community-based study in Sri Lanka was 6.3% among women aged between 15 and 39 years.^18^ The variation in the results of this study and the previous studies may be attributed to differences in age-groups, ethnic backgrounds, lifestyle factors, and the use of different diagnostic criteria.

The prevalence of infrequent menstruation in our sample was 36.7%, whereas the prevalence of frequent menstruation was 25.2%. A previous study on young adult Korean females found that among the 462 participants, 0.9% experienced frequent menstruation (<21 days) and 6.1% experienced infrequent menstruation (>35 days).^19^ Another study on 170 Indian women of reproductive age reported a 68% prevalence of menstrual irregularities.^11^ These discrepancies in the magnitudes of estimates in this study and reported by the previous studies regarding the prevalence of menstrual irregularities could be attributed to differences in age of the study participants, lifestyles and environmental factors.

Among the study participants, the prevalence of hirsutism, acne, male pattern baldness, difficulty in losing weight, and discoloration was 32.3%, 74.0%, 26.4%, 43.0%, and 39.6%, respectively. A previous study reported that 37.0% of African American females of reproductive age reported being bothered by excess hair, and 10% had hirsutism diagnosed based on the modified Ferriman-Gallwey pictorial assessment.^20^ The difference in the prevalence of hirsutism may be due to the fact that this study assessed the hirsutism based on the self-report without checking the participants’ medical records. The prevalence of acne in our study was 74.0%, which is consistent with the results of a Malaysian study, which reported 75.8% prevalence of acne among high school and university students.^21^ On the contrary, in a meta-analysis of 25 Chinese studies (83,008 subjects), the overall prevalence of acne across all age-groups was 10.2%.^22^ These results are quite variable because of different settings, contrasting age-groups, and diagnostic assays used. Moreover, an earlier study in India showed that the prevalence of male pattern baldness and discoloration in a randomly selected population of females in reproductive age was 25.0% and 30.0%, respectively, which is similar to the results of this study.^11^

The multivariable logistic regression model showed that the respondents have had significantly higher odds of having PCOS, if reportedly they had hyperprolactinemia. Recently, a study in this region has reported hyperprolactinemia as a frequent (37%) observation among patients with PCOS.^23^ Furthermore, it has been reported that a pathological elevation of the circulating prolactin level may result from prolactin adenomas, hyperprolactinemic drugs, and macroprolactin or could be an idiopathic hyperprolactinemia.^24,25^ However, further etiological studies are needed to find out the specific reasons of hyperprolactinemia to minimize the burden of PCOS.

The participants were more likely to have been diagnosed with PCOS if they suffered from menstrual irregularities. In India, a cohort study on 15- to 17-years-old participants reported significantly higher PCOS risk among those with confirmed menstrual irregularity as opposed to those without menstrual irregularity.^26^ This ill-health issue among the young adult segment of the population should be timely addressed and should call for the attention of public health practitioners.

In addition, this study found that the participants with a family history of PCOS have had significantly higher odds of having PCOS. This finding is congruous with the results reported previously wherein patients with PCOS had significantly higher proportions of mothers and/or sisters with PCOS than those without PCOS themselves.^27^

The final multivariable logistic regression model showed that the participants who reported experiencing hirsutism had higher odds of having PCOS. It has been shown previously that compared with the women without PCOS, among women with PCOS, diagnosis of hirsutism had higher frequency, while the two groups of women were nearly similar with respect to age, body mass index, oral contraceptive usage, acne, and menstrual pattern.^28^ Furthermore, the presence of hirsutism and menstrual irregularities had been regarded as the cardinal signs of PCOS.^29^

Moreover, our study revealed that women with male pattern baldness were more likely to have had PCOS diagnosis than women without male pattern baldness. Only a few reports have specifically examined the male pattern baldness (also known as androgenic alopecia) in women with PCOS. Like the finding of this study, a study from Turkey found a much higher prevalence (34.7%) of androgenic alopecia in women with PCOS compared with women without it.^30^ A case–control study found that the women with PCOS had a higher prevalence of male pattern baldness than the control participants; therefore, underscoring the fact that most women who present with androgenic alopecia as their primary complaint may also suffer from PCOS.^31^

### Strengths and limitations

There are some notable strengths of this study. First, the study sample comprised participants who were homogenous regarding education, as they were all university students. Second, to the best of our knowledge, this is the first study that assessed the prevalence of and risk factors associated with PCOS in young female population in Kuwait and provided requisite baseline information for future research.

Some limitations should be taken into consideration while interpreting the results. First, this was a cross-sectional study; therefore, temporal relationship between PCOS and the studied exposures cannot be established. Second, data were collected using a self-administered questionnaire with chances of recall bias*.* Third, this study only measured the self-report of physicians-diagnosed PCOS without checking the participants’ medical records, which might have resulted in some inconsistencies. However, likelihood of such reporting errors is believed to be minimal considering education type and level in the study sample. Fourth*,* women in our sample might not have been equally knowledgeable and aware of PCOS as has been shown elsewhere.^32^ Therefore, owing to poor knowledge and unawareness of the PCOS among the participants, the prevalence of PCOS might have been somewhat underestimated. Finally, another notable limitation of our study was that we used menstruation irregularities as one of the symptoms of PCOS. This symptom perhaps was not clearly defined in the study instrument and seemingly was not uniformly understood by the participants. Consequently, the prevalence of menstruation irregularities was very low among the participants with PCOS as opposed to those who reportedly were without PCOS. Future studies may consider dealing with this anomaly in the study instrument at a planning stage.

## Conclusions

In summary, this study recorded a moderately high prevalence (16.3%) of PCOS among female university students. Furthermore, hyperprolactinemia, menstrual irregularities, family history of PCOS, and male pattern baldness were identified as the significant risk factors for PCOS in the study population. Imparting awareness about this ill-condition and encouragement of women who suffer from PCOS-related symptoms to seek timely medical care may help minimizing the magnitude of this ailment and related psychological burden in this and other similar settings in the region. If implemented, future studies may look at the impact of such efforts.

## Data Sharing Statement

Deidentified individual participant data will not be made available.

## Abbreviations Used

aOR: Adjusted odds ratio
CI: Confidence interval
NIH: National Institute of Health
OR: Odds ratio
PCOS: Polycystic ovary syndrome
